# Mitochondrial DNA mutations can influence the post-implantation development of human mosaic embryos

**DOI:** 10.3389/fcell.2023.1215626

**Published:** 2023-08-11

**Authors:** Akifumi Ijuin, Hiroe Ueno, Tomonari Hayama, Shunsuke Miyai, Ai Miyakoshi, Haru Hamada, Sumiko Sueyoshi, Shiori Tochihara, Marina Saito, Haruka Hamanoue, Teppei Takeshima, Yasushi Yumura, Etsuko Miyagi, Hiroki Kurahashi, Hideya Sakakibara, Mariko Murase

**Affiliations:** ^1^ Reproduction Center, Yokohama City University Medical Center, Yokohama, Kanagawa, Japan; ^2^ Department of OB and GYN, Yokohama City University School of Medicine Graduate School of Medicine, Yokohama, Kanagawa, Japan; ^3^ Department of GYN, Yokohama City University Medical Center, Yokohama, Kanagawa, Japan; ^4^ Division of Molecular Genetics, Institute for Comprehensive Medical Science, Fujita Health University, Toyoake, Aichi, Japan; ^5^ Department of Clinical Genetics, Faculty of Medicine, Yokohama City University, Yokohama, Kanagawa, Japan

**Keywords:** mosaic embryo, mitochondria, mitochondrial DNA mutation, aneuploid cell decrease, post-implantation development

## Abstract

**Introduction:** Several healthy euploid births have been reported following the transfer of mosaic embryos, including both euploid and aneuploid blastomeres. This has been attributed to a reduced number of aneuploid cells, as previously reported in mice, but remains poorly explored in humans. We hypothesized that mitochondrial function, one of the most critical factors for embryonic development, can influence human post-implantation embryonic development, including a decrease of aneuploid cells in mosaic embryos.

**Methods:** To clarify the role of mitochondrial function, we biopsied multiple parts of each human embryo and observed the remaining embryos under *in vitro* culture as a model of post-implantation development (*n* = 27 embryos). Karyotyping, whole mitochondrial DNA (mtDNA) sequencing, and mtDNA copy number assays were performed on all pre- and post-culture samples.

**Results:** The ratio of euploid embryos was significantly enhanced during *in vitro* culture, whereas the ratio of mosaic embryos was significantly reduced. Furthermore, post-culture euploid and culturable embryos had significantly few mtDNA mutations, although mtDNA copy numbers did not differ.

**Discussion:** Our results indicate that aneuploid cells decrease in human embryos post-implantation, and mtDNA mutations might induce low mitochondrial function and influence the development of post-implantation embryos with not only aneuploidy but also euploidy. Analyzing the whole mtDNA mutation number may be a novel method for selecting a better mosaic embryo for transfer.

## 1 Introduction

*In vitro* fertilization and embryo transfer (IVF-ET) are the ultimate therapies for patients with infertility, capable of achieving higher pregnancy rates than other approaches. Embryonic euploidy is the most critical factor governing live births. In assisted reproductive technology (ART), aneuploidy was found to be responsible for approximately 70% of miscarriages (Kato et al., 2022) and unsuccessful transfers. Considering euploid embryos diagnosed using pre-implantation genetic testing for aneuploidy (PGT-A), the implantation rate per transfer reached 70%. In contrast, the implantation rate per transfer without PGT-A was less than 40% and gradually decreased with maternal age (Simon et al., 2018; Sato et al., 2019).

Recent developments in genetic testing technologies have enabled the determination of detailed chromosomal information of embryos, thereby revealing the existence of mosaic embryos that include both euploid and aneuploid blastomeres. Mosaic embryos may account for 23–33% of blastocysts (Fragouli et al., 2019). Although mosaic embryos are considered a type of aneuploid embryo, clinical outcomes of mosaic embryos were better than those of aneuploid embryos, with a live birth rate of 33.3%, and all newborns were healthy (Greco et al., 2015). Several studies have reported similar results (Munné et al., 2017; Spinella et al., 2018; Fragouli et al., 2019). Accordingly, mosaic embryos can be considered for transfer. We need to select efficient mosaic embryos with high potential to develop into healthy newborns. Whereas methods to classify mosaic embryos are yet to be established, because the background of healthy newborns from mosaic embryos remains unclear. Therefore, we aimed to reveal this background and develop a method to classify mosaic embryos. A mouse embryo study has shown that aneuploid cells in mosaic embryos progressively decrease during embryonic development (Bolton et al., 2016) and that aneuploid cells were eliminated during cell-proliferation competition at the post-implantation stage (Hashimoto and Sasaki, 2020). The underlying factor responsible for this decrease in aneuploid cells is yet to be identified, and we hypothesized that certain factors are also critical for the development of human mosaic embryos.

Mitochondria are organelles in eukaryotic cells responsible for cellular energy demands, such as ATP with oxidative phosphorylation (OXPHOS) (May-Panloup et al., 2021). Given that the main cellular energy for blastocysts is supplied by drastically upregulated OXPHOS and glycolysis, and embryonic development requires biological energy, mitochondrial function is an important factor in embryonic development. Established methods require a large number of cells to directly analyze mitochondrial function. A cell contains hundreds of mitochondria, and a mitochondrion contains many copies of mitochondrial DNA (mtDNA). In the case of embryo analysis, only a few cells are available from an embryo. mtDNA is the only factor used to evaluate mitochondrial quantity and function in the embryo, and sufficient mtDNA can be collected from a few cells. Based on the hypothesis that mitochondrial function is regulated by mtDNA quantity (Williams, 1986; Hock and Kralli, 2009), several previous studies have examined the correlation between mtDNA quantity and embryonic development, revealing ambiguous findings (Podolak et al., 2022b). Each mtDNA molecule includes mutations that occasionally induce dysfunction owing to the location of the mutation and the percentage of mutated mtDNA, called heteroplasmy. In embryo research, detailed mtDNA mutation analysis is used to determine mitochondrial function from mtDNA. We focused on the mtDNA mutation number, as well as additional factors such as heteroplasmy and location.

To reveal the correlation between mitochondrial function and post-implantation embryonic development, it is important to evaluate mtDNA copy number and mtDNA mutations, which can induce mitochondrial dysfunction (Cecchino and Garcia-Velasco, 2019). In the present study, we aimed to clarify the correlation between mitochondria and post-implantation embryonic development, including the development of mosaic embryos into euploids, and the difference between well-developed post-implantation embryos and other embryos. To meet this objective, we examined changes in karyotype, mtDNA copy number, and mtDNA mutations in embryos from the blastocyst stage to the *in vitro* artificial post-implantation stage.

## 2 Material and methods

### 2.1 Experimental design

In this study we aimed to evaluate the karyotype and mitochondria at peri-implantation stage. Firstly, to collect samples at pre-implantation stage, we biopsied 1–3 parts of trophectoderm from each donated embryo. In addition, to obtain samples at post-implantation stage, we performed *in vitro* culture as model of implantation and collected some parts of proliferated cell mass.

We performed karyotyping to each sample, in order to evaluate the change of embryonic karyotype during post-implantation development. To clarify the role of mitochondrial quantity and quality during post-implantation development of human embryos, we analyzed mtDNA mutations and mtDNA copy number. Karyotyping and mtDNA mutation analysis was performed with next generation sequencing, and mtDNA analysis was performed with real time PCR.

### 2.2 Ethical review

This study was approved under the ethics committee of Yokohama City University (approval number: A171130002), and the Japan Society of Obstetrics and Gynecology (approval number: 192).

### 2.3 Subjective embryos

This study included not only morphologically high-grade surplus embryos but also degraded embryos to evaluate the culture process of mosaic embryos. All embryos were created initially for clinical use in ART at our institution. They were donated because the patients achieved live birth with another embryo, or because the embryos were not used clinically due to slow development or morphologically low grade. All patients provided a written informed consent before donation and research use.

### 2.4 Embryo biopsy

**FIGURE 1:**
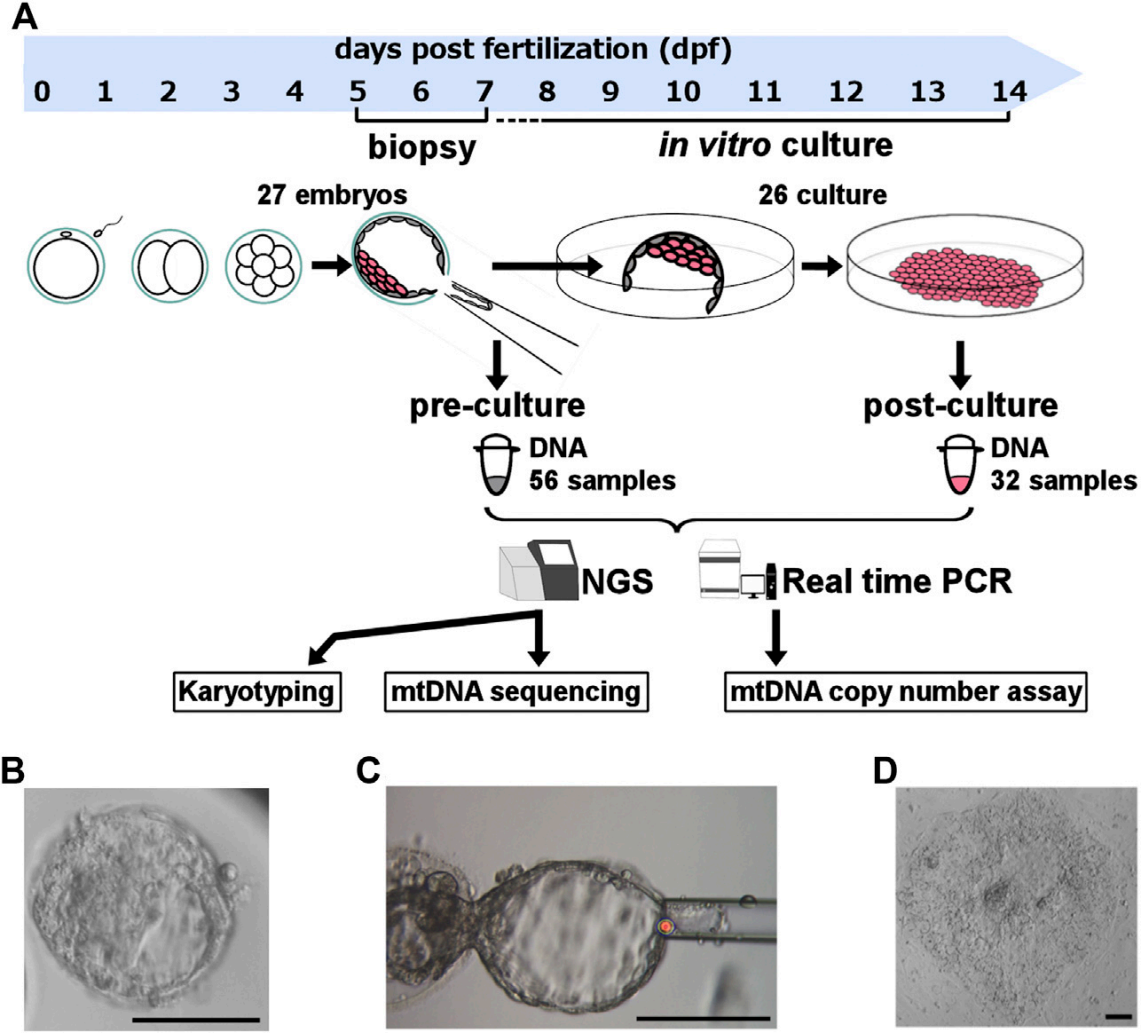
Experimental design. **(A)** Experimental design and sample size. To clarify the role of mitochondrial quantity and quality during the post-implantation stage of human embryos, we biopsied the trophectoderm from donated human embryos, performed *in vitro* culture for artificial implantation, and analyzed the karyotype and mitochondrial DNA sequence with NGS and mtDNA copy number using real-time PCR. **(B)** Morphological findings of experimental blastocysts before biopsy. **(C)** Morphological findings of blastocysts obtained during biopsy. **(D)** Morphological findings of experimental cell mass 4‒5 days post-biopsy culture. The scale bar indicates 100 μm. mtDNA, mitochondrial DNA; NGS, next-generation sequencing.

On 5–7 day post-fertilization (dpf), all blastocysts were biopsied one to three times from TE with laser pulse and quick flicking movement, avoiding ICM injury (Figures 1A–C). These biopsied TE samples (pre-culture sample) contained 5–10 cells each. If the biopsied embryos did not shrink after TE biopsy, additional biopsies were performed. In contrast, when embryos shrank after TE biopsy, we did not perform additional biopsy to avoid too much embryo damage for subsequent culture. To prepare for post-biopsy culture, we mechanically removed zona pellucida from the remained embryo, and cells adhering to zona pellucida were collected as TE (pre-culture sample). All pre-culture samples were washed and applied to the reagent for DNA extraction.

### 2.5 *In vitro* culture modelling post-implantation development

The remained embryo containing ICM were immediately plated and cultured individually per plate (Figure 1A). The *in vitro* culture modelling post-implantation development started with a medium including the following ingredients as previously described (Tachibana et al., 2013): Knockout DMEM/F12 (Gibco, Cambridge, United Kingdom), 10% Knockout Serum Replacement (Gibco, Cambridge, United Kingdom), 10% FBS, 1% Penicillin-Streptomycin-Glutamine (Fujifilm, Tokyo, Japan), 1% Non-Essential Amino Acid (Gibco, Cambridge, United Kingdom), 0.1 mM β-Mercaptoethanol (Sigma-Aldrich, St. Louis, US), 5 ng/mL basic Fibroblast Growth Factor (Fujifilm, Tokyo, Japan), and 10 μM Rock Inhibitor (Enzo Life Sciences, NY, United States). We removed the Rock Inhibitor and FBS and added knockout serum replacement to 20% after outgrowth occurred.

Tachibana, who referred to the culture method used in this present study, described that the expression of Oct-4 and Sox-2 was confirmed in the proliferated cell mass by immunostaining, and the images were shown (Tachibana et al., 2013). Based on this report, we thought the outgrowth of the embryos in this *in vitro* culture can contain the cells derived from ICM, and we analyzed its outgrowth as grown embryos like post-implantation stage.

We checked the horizontal and vertical cell proliferation everyday by multi-focus observation under microscope. This culture was performed until 13 dpf with ethical permission, and the maximum culture period was 7 days. The days until cell proliferation completely stopped were defined as “culture period.” We defined the embryos that were alive at 7th day after biopsy as “culturable embryos”, meaning they achieved post-implantation stage in our *in vitro* culture. At the final day of culture, we collected part of cell mass that are post-culture samples (Figure 1D). Dead cells may be possibly included in post-culture samples, but they are not thought to effect karyotype and mtDNA. Because scale of cells was larger in post-culture sample than embryos’, we were able to evaluate them correctly from post-culture samples.

### 2.6 DNA extraction

DNA was extracted with a Pico Pure DNA extraction kit (Applied Biosystems Inc., Foster City, CA, United States) from all samples individually with modified manufacturer’s protocol. The DNA solution was diluted with DNA RNA free water and used for karyotyping, mtDNA copy number assay, and mtDNA mutation assay.

### 2.7 Karyotyping

Karyotyping of all samples was performed at OVUS Co., Ltd. (Aichi, Japan). Whole genome amplification was performed using the SurePlex WGA Kit (Illumina, San Diego, CA) according to the manufacturer’s protocol. Nextera libraries were prepared from the amplified DNA and subsequently sequenced with a VeriSeq PGS assay system by MiSeq (Illumina, San Diego, CA). The sequencing data were analyzed by BlueFuse Multi analysis software v4.5. The karyotype of each embryo at pre- and post-culture was determined as “euploid,” “mosaic,” or “full aneuploid.” Because single TE biopsy is not sufficient to decide correct karyotype of embryo (Takahashi et al., 2021), our decision was based on their uniformity when multiple samples were simultaneously collected from the same embryo. When all results were “euploid” or the same “aneuploidy,” the embryo was determined as “euploid” or “full aneuploid.” When they did not match, the embryo was determined as “mosaic.” When the mosaicism rate was between 20% and 80% for a single sample, karyotype of the sample was determined to be mosaic. We calculated the ratios of each karyotype at pre- and post-culture separately to evaluate the change of karyotype during *in vitro* culture.

### 2.8 Relative mitochondrial DNA (mtDNA) copy number assay

The Mitochondrial DNA (mtDNA) copy number of each embryo were quantified by relative real-time polymerase chain reaction (PCR) assay as previously described (Fragouli et al., 2017) to normalize data within samples. We used SYBR Green detection (Applied Biosystems Inc., Foster City, CA, United States) on a 7900-HT Real-Time PCR System (Applied Biosystems Inc., Foster City, CA, United States) (Figure 1A) with modified manufacturer’s protocol. The real-time PCR was performed in duplicates because of little amount DNA samples. The mean value was obtained. When the dissociation curve was inappropriate, the corresponding well data was not included in the study. We evaluated the β2-microgrobulin gene (*B2M*) on nuclear genome as an endogenous standard, and the tRNA of leucine gene on mtDNA (*MT-TL1*), and calculated the difference of the Ct value (∆Ct). Then we supposed that 100 cells were included in 1 blastocyst and calculated the mtDNA copy number included in each embryo by 100 × 2^−ΔΔCT+1^. Based on previous report (Hashimoto et al., 2017), we excluded data that resulted in measurements of more than 1 × 10^6^ copies per embryo as measurement errors.

### 2.9 mtDNA mutation assay

Firstly, we selectively amplified the mtDNA by long-range PCR for two overlapping amplicons (8106 bp and 8609 bp) using TaKaRa LA Taq Hot Start Version (TaKaRa Biomedicals, Otsu, Japan) with modified manufacturer’s protocol. Sequencing was performed at OVUS Co., Ltd. (Aichi, Japan). Nextera XT DNA Library Preparation Kit (Illumina, San Diego, CA, United States) was used to fragment the amplified mtDNA following the manufacturer’s instructions. Then, AMPure XP (Beckman Coulter, Pasadena, CA, United States) was used to purify the library DNA and to remove short library fragments. Sequencing of the DNA samples were performed using Illumina MiSeq (Figure 1A).

The mtDNA sequence was compared to a reference sequence (revised Cambridge Reference Sequence; rCRS, GenBank number: NC_012920) using MITOMASTER, which is the part of mtDNA database MITOMAP. MITOMAP includes mtDNA nucleotide variants from human mtDNA sequences from the GenBank dataset. The MITOMASTER provides mtDNA variation, including variant frequency, and haplogroup determination (Lott et al., 2013). Mitochondrial haplogroup were identified by MITOMASTER, and the total number and location of all variants for each samples were obtained. Next, we excluded haplogroup marker (present at frequency of 80% or higher in that specific haplogroup (McCormick et al., 2020)). As for coding gene, mutations that cause amino acid changes, and achieved ≥90% heteroplasmy were included in this analysis. This is because ≥90% heteroplasmic mtDNA point mutations can generally worsen mitochondrial activity (Podolak et al., 2022b). As for tRNA mutations with ≥90% heteroplasmy, we included likely pathogenic mutations defined by MitoTIP tool on MITOMAP, and pathogenic mutations confirmed in MITOMAP. We calculated heteroplasmy as the percentage of reads that detected each mutation among all reads at the same detection site. We evaluated the number of these high heteroplasmic and non-synonymous mutations in embryo. When multiple samples were simultaneously collected from the same embryo, the number of mtDNA mutations in each embryo was adjusted by the number of samples as below. We counted the number of mutations per embryo by adding the number of mtDNA mutations on samples of each embryo and dividing it by the number of the samples. For example, in case of embryo No. 1, number of pre- and post-culture mutations were calculated as 0, because sample No. 1-1, 1–2, 1–3, and 1-4 had no ≥ 90% heteroplasmic and non-synonymous mutation. Therefore, number of mutations was calculated to be 0. In case of embryo No. 10, all samples (2 pre-culture samples and 3 post-culture samples) had one heteroplasmy ≥90% mutation each. Number of mutations at pre-culture stage was calculated as (1 + 1)/2, and that of post-culture stage was calculated as (1 + 1+1)/3. Therefore, embryo No.10 had one mutation at both of pre- and post-culture stage.

### 2.10 Statistical analysis

The percentage data and means were compared with Fisher’s exact test and two-sided student t-test. All statistical analyses were performed with EZR (Saitama Medical Center, Jichi Medical University, Saitama, Japan), a graphical user interface for R (The R Foundation for Statistical Computing, Vienna, Austria). It is a modified version of the R commander designed to add statistical functions frequently used in biostatistics (Kanda, 2013). A value of *p* < 0.05 was chosen as statistical significance.

## 3 Results

### 3.1 Subjects and donated embryos

We recruited 17 patients with infertility at Yokohama City University Medical Center. The study was approved by Yokohama City University. The mean patient age was 35.6 years. In total, 27 blastocysts were analyzed, most of which were morphologically evaluated as low-grade (Gardner grade BB, 30%; CB, 11%; CC, 59%, Table 1).

**TABLE 1:** Summary of results from each embryo (culture, karyotype and mtDNA analysis).

Embryo no.	Embryo status at biopsy	Culturability (final day of culture)	Karyotyping (pre-culture > post-culture)	Mean mtDNA copy number			Raw number of mtDNA mutation in all samples (pre-culture > post-culture)	Mean mtDNA mutation number		
				Pre-culture sample	Post-culture sample	Trend		Pre-culture sample	Post-culture sample	Trend
1	CB (7dpf)	culturable (14dpf)	euploid > euploid	372395	75797	↓	0, 0, 0 > 0	0.0	0.0	→
2	CB (7dpf)	culturable (14dpf)	euploid > euploid	75232	95808	↑	0, 0 > 0	0.0	0.0	→
3	BB (5dpf)	non-culturable (7dpf)	mosaic > euploid	ND	17865	ND	ND, ND > 1	ND	1.0	ND
4	CC (6dpf)	non-culturable (11dpf)	mosaic > euploid	13624	238537	↑	0, 0, 0 > 0, 0	0.0	0.0	→
5	CC (6dpf)	non-culturable (10dpf)	mosaic > euploid	539604	88898	↓	2, 2, 2 > 2	2.0	2.0	→
6	CC (7dpf)	culturable (14dpf)	mosaic > euploid	131788	35764	↓	0, 0, 0 > 0, 0, 0	0.0	0.0	→
7	BB (7dpf)	culturable (14dpf)	mosaic > euploid	124033	94948	↓	0, 0, 0 > 0	0.0	0.0	→
8	CC (7dpf)	culturable (14dpf)	mosaic > euploid	130601	71609	↓	0, 0, 0 > 0	0.0	0.0	→
9	BB (6dpf)	culturable (13dpf)	mosaic > euploid	190039	118527	↓	0, 0, > 0	0.0	0.0	→
10	CC (6dpf)	culturable (13dpf)	mosaic > euploid	57763	42677	↓	1, 1 > 1, 1, 1	1.0	1.0	→
11	CC (6dpf)	culturable (13dpf)	mosaic > euploid	92608	105571	↑	0, 0 > 0	0.0	0.0	→
12	CC (6dpf)	culturable (13dpf)	mosaic > euploid	ND	70918	ND	1, 0 > 1	0.5	1.0	↑
13	CC (6dpf)	culturable (13dpf)	mosaic > euploid	ND	96140	ND	0 > 0	0.0	0.0	→
14	CC (6dpf)	culturable (13dpf)	mosaic > euploid	ND	196321	ND	0 > 0	0.0	0.0	→
15	CC (7dpf)	culturable (14dpf)	euploid > mosaic	181405	38109	↓	1 > 1	1.0	1.0	→
16	CC (7dpf)	culturable (14dpf)	euploid > mosaic	132612	47408	↓	1 > 1	1.0	1.0	→
17	BB (5dpf)	non-culturable (7dpf)	mosaic > mosaic	ND	ND	ND	ND > ND	ND	ND	ND
18	BB (6dpf)	non-culturable (12dpf)	mosaic > mosaic	ND	ND	ND	0, 1, 0 > 0	0.3	0.0	↓
19	CC (6dpf)	non-culturable (10dpf)	mosaic > mosaic	ND	70918	ND	2, 2 > 2	2.0	2.0	→
20	CB (6dpf)	non-culturable (12dpf)	full aneuploid > mosaic	ND	65167	ND	2 > 2	2.0	2.0	→
21	CC (6dpf)	non-culturable (9dpf)	mosaic > full aneuploid	169696	554895	↑	2, 2, 2 > 2	2.0	2.0	→
22	CC (6dpf)	culturable (13dpf)	full aneuploid > full aneuploid	19496	45258	↑	2, 2, > 2, 2	2.0	2.0	→
23	CC (6dpf)	culturable (13dpf)	full aneuploid > full aneuploid	488790	86767	↓	1 > 1	1.0	1.0	→
24	BB (7dpf)	culturable (14dpf)	full aneuploid > full aneuploid	187153	85040	↓	0, 0 > 0	0.0	0.0	→
25	BB (6dpf)	not cultured	mosaic > ND	87217	ND	ND	0, 0 > ND	0.0	0.0	→
26	BB (6dpf)	non-culturable (12dpf)	mosaic > ND	252566	ND	ND	0, 0, 0 > ND	0.0	ND	ND
27	CC (7dpf)	culturable (14dpf)	full aneuploid > ND	76855	ND	ND	1 > 1	1.0	1.0	→

ND means “not determinable”. Listed mtDNA mutations are non-synonymous and heteroplasmy 90% or more.

### 3.2 TE biopsy and post-biopsy *in vitro* culture

TE biopsy was performed on all the 27 embryos 1–3 times per embryos. In total, 56 pre-culture samples (2.1 ± 0.8 samples per embryo [mean ± standard deviation (SD)]) were obtained.

The 26 post-biopsy embryos were plated and cultured. *In vitro* cultures were performed by 12.3 dpf on average (6–14 dpf), which was equivalent to 6.0 days post-biopsy (2–7 days). Seventeen culturable embryos (63.0% of cultured embryos) were available. Non-culturable embryos were cultured by 10.0 dpf on average, which was equivalent to 4.2 days post-biopsy. We collected 1-2 obtained 32 post-culture samples (1.2 ± 0.6 samples per embryo [mean ± SD]) were subjected to karyotyping, mtDNA sequencing, and mtDNA copy number assay. Supplementary Table S1 lists the results for all samples.

### 3.3 Decrease of mosaic embryos was observed during *in vitro* culture

To confirm the non-uniform development of mosaic embryos, we compared the karyotypes of 27 embryos before and after culturing (Figure 1A). The success rate for data acquisition of each sample were 89.3% (50/56) for pre-culture samples, and 90.6% (29/32), for post-culture samples. The success rate of embryo karyotyping was 100% (27/27) and 92% (24/26) for pre-culture and post-culture stage, respectively.

**FIGURE 2:**
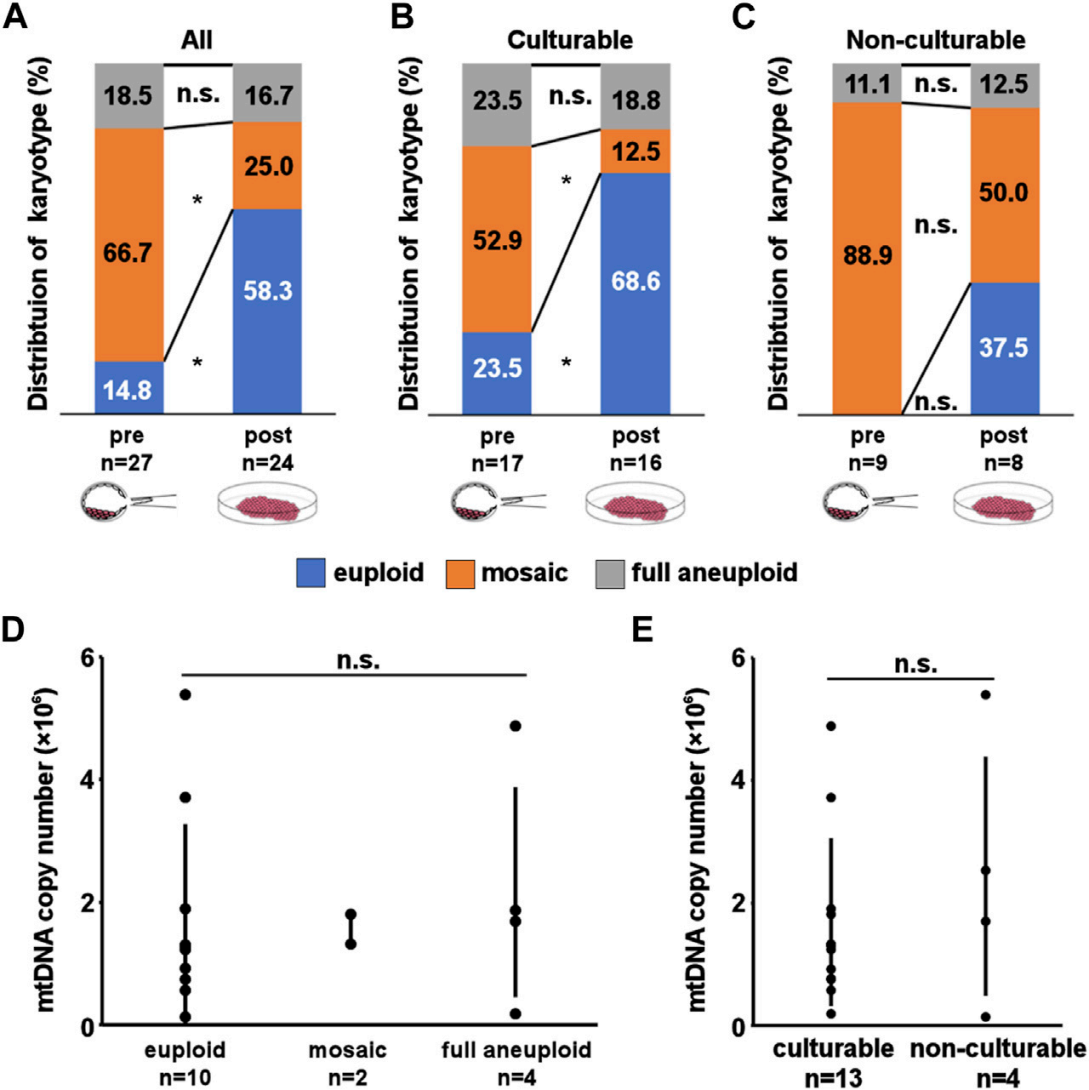
Result of Karyotyping and mitochondrial DNA (mtDNA) copy number assay. **(A–C)** Distribution of karyotypes in pre- and post-culture embryos. **(A, B, C)** show karyotype changes in all experimental, culturable, and non-culturable embryos, respectively. A significant increase in euploid embryos and a significant decrease in mosaic embryos can be observed in **(A, B)**. **(D, E)** mtDNA copy number in pre-culture samples. D shows the comparison of copy number between each post-culture karyotypes. The mtDNA copy number of each karyotype were 172769 ± 153311 for euploid, 157009 ± 24396 for mosaic, and 216284 ± 170297 for full aneuploid. E shows comparison of copy number between each culturability. Although culturable embryos had lower number of mtDNA copy number, the mean mtDNA copy number does not differ significantly between the groups. The mtDNA copy number of each culturability were 159509 ± 126220 for culturable embryos, 243872 ± 191082 for non-culturable embryos. mtDNA copy number is presented as mean ± SD. “*” represents *p* < 0.05. “n.s.” represents not significant. The bars in D and E represent error bar.

Table 1 and Supplementary Table S1 summarize karyotype distribution in all embryos. At pre-culture, 14.8% (4/27) were euploid, 66.7% (18/27) were mosaic, and 18.5% (5/27) were full aneuploid. At post-culture, 58.3% (14/24) were euploid, 25.0% (6/24) were mosaic, and 16.7% (4/24) were full aneuploid. After *in vitro* culture, the rate of euploid embryos significantly increased (*p* = 0.002), while that of mosaic embryos significantly decreased (*p* = 0.005) (Figure 2A). In contrast, no significant changes in the distribution of full aneuploid embryos were observed. The same trends were observed for culturability, with a significant change observed only in culturable embryos (euploid: *p* = 0.01, culturable embryo; *p* = 0.08, non-culturable embryo; mosaic: *p* = 0.03, culturable embryo; *p* = 0.13, non-culturable embryo; Figures 2B, C). These trends did not correlate with indications for ART or female age.

### 3.4 Post-implantation embryonic development is individual from mtDNA copy number

To clarify the underlying mechanism involved in post-implantation development, we analyzed the mtDNA copy number (Figure 1A). The success rate for data acquisition of each sample were 53.6% (30/56) for pre-culture samples, and 81.3% (26/32) for post-culture samples. We compared the results of pre-culture embryos between each post-culture karyotype (the number of determinable embryos was 10 euploids (embryo No. 1, 2, and 4–11), 2 mosaics (embryo No. 15 and 16), and 4 full aneuploids (embryo No. 21–24)) and culturability (the number of determinable embryos was 13 culturable embryos (embryo No. 1, 2, 6–11, 15, 16, and 22–24) and 4 non-culturable embryos (embryo No. 3–5 and 21)). Although culturable embryos had lower number of mtDNA copies, no significant correlations were observed (Figures 2D, E; Supplementary Table S2, S3). These results suggest that a simple difference in the mtDNA copy number is not critical for post-implantation development.

### 3.5 Analyzing the number of non-synonymous mtDNA mutations

For mtDNA mutation analysis, we selectively amplified mtDNA from all 88 samples derived from 27 embryos using polymerase chain reaction (PCR) and performed mtDNA sequencing via next-generation sequencing. The success rate for data acquisition of each sample were 92.9% (52/56) for pre-culture samples, and 96.9% (31/32) for post-culture samples. Supplementary Table S1 lists mutations for each sample with a heteroplasmy level of ≥90%. A total of 11 mutations were identified. The mean number of mtDNA mutations per embryo with a heteroplasmy level of more than 90% were 0.6 ± 0.8 (mean ± SD). The number of mutations in each embryo was unaltered between pre- and post-culture.

### 3.6 The number of mtDNA mutations was associated with post-implantation embryonic development

We determined the number of mtDNA mutations to examine whether this number was related to post-culture karyotype (13 euploid embryos (embryo No.1, 2, and 4–14) vs. 9 aneuploid embryos (including mosaic and full aneuploids, embryo No.15, 16, and 18–24)) and culturability (17 culturable embryos (embryo No. 1, 2, 6–16, 22–24, and 27) vs. 7 non-culturable embryos (embryo No. 4, 5, 18–21, and 26)) and clarify the influence of mtDNA mutations on post-implantation development. In our model, the post-culture status can be considered as model of post-implantation status. We evaluated the mean number of mtDNA mutations that achieved ≥90% heteroplasmy, given that these mtDNA point mutations generally worsen mitochondrial activity. The number of mtDNA mutations is presented as mean ± SD.

**FIGURE 3:**
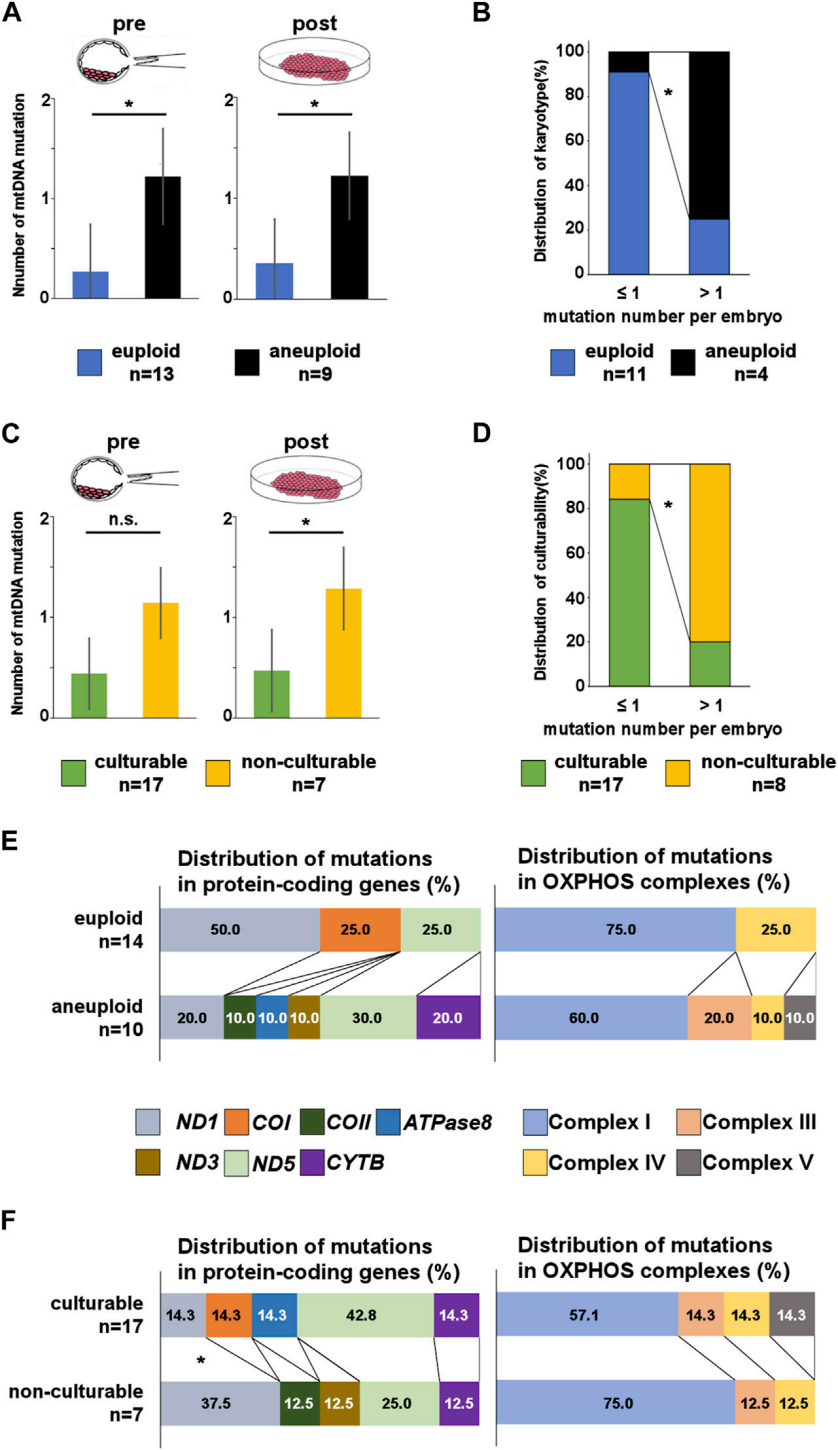
Number and location of mtDNA mutations in pre- and post-culture embryos. **(A, C)** Number of mutations per embryo in each post-culture karyotype and culturability at the pre- and post-culture stages. Post-culture euploid embryos exhibit a significantly lower number of mutations than post-culture aneuploid embryos at both pre- and post-culture stages. The number of mutations is lower in culturable embryos than in non-culturable embryos at both pre- and post-culture stages. **(B)** Comparing the distribution of post-culture karyotype between pre-culture mosaic embryos with lower number of mtDNA mutations and pre-culture mosaic embryos with higher number of mtDNA mutations. **(D)** Comparing the distribution of culturability between embryos with lower number of mtDNA mutations and embryos with higher number of mtDNA mutations. **(E, F)** Distribution of mtDNA mutations in protein-coding genes and OXPHOS complexes. C shows that mutations in *COII, ATPase8, ND3, CYTB*, and complexes III and V are more frequent in post-culture aneuploid embryos. D shows that mutations in *ND1, COII* and *ND3* are more frequent in non-culturable embryos. “*” represents *p* < 0.05. “n.s.” represents not significant. mtDNA, mitochondrial DNA. The bars in A and B represent error bar.

Euploid embryos had a significantly low number of pre-culture mtDNA mutations (euploid vs. aneuploid: 0.27 ± 0.57 vs. 1.22 ± 0.66, *p* = 0.005; Figure 3A). Moreover, a similar trend was observed for post-culture mtDNA mutations (euploid vs. aneuploid: 0.36 ± 0.61 vs. 1.22 ± 0.79, *p* = 0.010; Figure 3A).

Next, we counted mtDNA mutations only in pre-culture mosaic embryos and compare the number between each post-culture karyotype. We found post-culture euploid embryos (*n* = 11: embryo No.4–14) had a lower number of mtDNA mutations than post-culture aneuploid embryos (*n* = 3: embryo No.18, 19, and 21) [euploid vs. aneuploid; 0.32 ± 0.60 vs. 1.33 ± 0.90 (*p* = 0.061, pre-culture sample), 0.42 ± 0.60 vs. 1.33 ± 0.90 (*p* = 0.86, post-culture sample)], suggesting mtDNA mutation number may function as a marker for predicting transfer outcomes with mosaic embryos. In addition, we evaluated about mosaic embryos with more than 1 pre-culture mtDNA mutation. The post-culture euploid embryos were only 1 in them, and this was significantly lower than embryos with under 1 pre-culture mtDNA mutation (*p* = 0.03, Figure 3B).

Furthermore, culturable embryos had a lower number of pre-culture mtDNA mutations than non-culturable embryos (culturable vs. non-culturable: 0.44 ± 0.59 vs. 1.14 ± 0.99, *p* = 0.053; Figure 3C). Culturable embryos had a significantly lower number of post-culture mtDNA mutations than non-culturable embryos (culturable vs. non-culturable: 0.47 ± 0.61 vs. 1.29 ± 0.88, *p* = 0.021; Figure 3C). These results suggest that the number of mtDNA mutations could be related to post-implantation embryonic development and a decrease in aneuploid cells in the embryo. In addition, we evaluate culturability of embryos with more than 1 pre-culture mtDNA mutation. The culturable embryos were only 1 in them, and this was significantly lower than embryos with under 1 pre-culture mtDNA mutation (*p* = 0.02, Figure 3D).

### 3.7 The location of mtDNA mutations was associated with post-implantation embryonic development

To clarify the role of each mtDNA gene, we determined the number and location of ≥90% heteroplasmic and non-synonymous mtDNA mutations to establish their potential relationship with post-culture karyotype and culturability (Table 2). The number of mtDNA mutations at each gene site was adjusted based on the number of pre-culture samples collected from each embryo. We also counted the number of embryos showing mtDNA mutation on each gene (Table 3). Herein, we detected mutations in seven mtDNA genes (*ND1*, *COI*, *COII*, *ATPase8*, *ND3*, *ND5*, and *CYTB*) in ten embryos. The *ND5* mutation was the most frequent per embryo at all locations (33.3%), followed by *ND1* and *CYTB* (26.7% and 13.3%, respectively). Embryos with *ND5* mutations were 5 (50%), embryos with *ND1* mutations were 4 (40%), embryos with *CYTB* mutation were 2 (20%), and embryos with other mutations were 1 (10%) for each gene.

**TABLE 2:** The number of mtDNA mutations per embryo.

Protein complex no.	Complex I				Complex III	Complex IV			Complex V
Gene name	*ND1*	*ND3*	*ND5*	total	*CYTB*	*COI*	*COII*	total	*ATPase8*
Total	0.17 ± 0.38	0.04 ± 0.20	0.25 ± 0.44	0.46 ± 0.71	0.08 ± 0.28	0.04 ± 0.20	0.04 ± 0.20	0.8 ± 0.27	0.04 ± 0.20
Culturable	0.06 ± 0.24	none	0.24 ± 0.44	0.29 ± 0.47	0.06 ± 0.24	0.06 ± 0.24	none	0.06 ± 0.24	0.06 ± 0.24
Non-culturable	0.43 ± 0.53	0.14 ± 0.38	0.29 ± 0.49	0.86 ± 1.07	0.14 ± 0.38	none	0.14 ± 0.38	0.14 ± 0.38	none
Euploid	0.14 ± 0.35	none	0.14 ± 0.35	0.29 ± 0.59	none	0.07 ± 0.26	none	0.07 ± 0.26	none
Aneuploid	0.20 ± 0.40	0.10 ± 0.30	0.10 ± 0.30	0.60 ± 0.84	0.20 ± 0.40	none	0.10 ± 0.30	0.10 ± 0.32	0.10 ± 0.30

**TABLE 3:** The number and rate of embryos which had mtDNA mutations on each gene.

Protein complex no.	Complex I				Complex III	Complex IV			Complex V
Gene name	*ND1*	*ND3*	*ND5*	total	*CYTB*	*COI*	*COII*	total	*ATPase8*
Culturable embryo (n = 17)	1 (6%)	0 (0%)	3 (18%)	4 (24%)	1 (6%)	1 (6%)	0 (0%)	1 (6%)	1 (6%)
Non-culturable embryo (N = 7)	3 (43%)	1 (14%)	2 (29%)	3 (43%)	1 (14%)	0 (0%)	1 (14%)	1 (14%)	0 (0%)
Euploid embryo (n = 14)	2 (14%)	0 (0%)	1 (7%)	2 (20%)	0 (0%)	1 (7%)	0 (0%)	1 (7%)	0 (0%)
Aneuploid embryo (n = 10)	2 (20%)	1 (10%)	3 (30%)	4 (29%)	2 (20%)	0 (0%)	1 (10%)	1 (10%)	1 (10%)

We investigated the correlation between pre-culture mtDNA mutations and post-culture karyotypes. Mutations in *COII*, *ATPase8*, *ND3*, and *CYTB* were detected only in the post-culture aneuploid embryos (Figure 3E), and the rate of each mutation were accounting for 10%, 10%, 10%, and 20% of all mtDNA mutations in post-culture aneuploid embryos, respectively. The number of *CYTB* mutations were the highest in all four genes. The number of embryos with *ND5* were 3 (30% of post-culture aneuploid embryos), embryos with CYTB were 2 (30% post-culture aneuploid embryos), and embryos with others were 1 for each (10% post-culture aneuploid embryos), respectively. Categorizing the locations of these mutations by the OXPHOS complex, mutations in the complex III gene (*CYTB*) and complex V gene (*ATPase8*) were found only in post-culture aneuploid embryos (Figure 3E). In embryos diagnosed as pre-culture mosaic embryos, only post-culture aneuploid embryos had mutations in *COII*, *CYTB* and complex III genes (*CYTB*).

Next, we investigated the correlation between pre-culture mtDNA mutations and culturability. The mean numbers of mutations in *ND1* were 0.06 ± 0.24 vs. 0.43 ± 0.53 (mean ± SD, culturable vs. non-culturable, *p* = 0.027), and mutations in *COII* and *ND3* were only detected in non-culturable embryos. Among all mutations, the rates of *ND1* mutations in culturable and non-culturable embryos were 14.3% and 37.5%, respectively. *COII* and *ND3* mutations accounted for 12.5% of all mutations in non-culturable embryos, and *ND1* mutations were most frequently present in these three genes (Figure 3F). Embryos with *ND1* mutation were 3 (43% of non-culturable embryos), and embryo with *COII* or *ND3* was 1 (10% of non-culturable embryos) for each. The number of *ND1*, *COII*, and *ND3* mutations had significantly higher frequencies in non-culturable embryos than in culturable embryos. However, the number of mutations in each OXPHOS complex did not differ significantly (Figure 3F).

## 4 Discussion

To the best of our knowledge, this is the first report to demonstrate a possible correlation between mtDNA mutations and human embryonic development. Certain mosaic embryos, as diagnosed by PGT-A at the blastocyst stage, have been found to develop into healthy babies (Greco et al., 2015). This could be attributed to the gradual decrease in aneuploid cells during mosaic embryonic development, which has been documented in mice (Bolton et al., 2016). We investigated that some mosaic blastocysts can develop as euploids within or around one-week post-implantation, leading to a healthy live birth. Accordingly, it can be suggested the decrease in aneuploid cells during human embryonic development. Furthermore, we hypothesized that mitochondria participate in post-implantation embryonic development, owing to their various roles in embryonic development (Podolak et al., 2022b). Our mtDNA analysis revealed that mtDNA mutations partly impacted the post-implantation development of human embryos, whereas mtDNA copy number exhibited no such impact.

Previously, a decrease in aneuploid cell number has been suggested in human embryos, although poorly clarified. In a mouse model, artificially obtained mosaic embryos showed that aneuploid cells were eliminated by more than 50% embryos within a few days post-implantation (3 or 4 days after blastocyst transfer) and were further eliminated or absent from the fetus in 66% of embryos at 13.5 dpf (10 days after blastocyst transfer) (Bolton et al., 2016). This finding indicates that abnormal cells are eliminated from mouse mosaic embryos during post-implantation development. Considering our findings on karyotype distribution, the proportion of mosaic embryos was significantly decreased (from 66.7% to 25.0%), while that of euploid embryos significantly increased (from 14.8% to 58.3%) during human embryo post-implantation culture. We concluded that some mosaic blastocysts can develop into euploids within approximately one-week post-implantation, suggesting that aneuploid cell numbers decrease during the development of human embryos. One study on the accuracy of PGT-A with TE biopsy using human embryos has compared the karyotypes of TE biopsy samples and post-*in vitro* culture cells. The authors concluded that there were biological and methodological difficulty in diagnosing mosaic embryos using a single TE biopsy, because of low concordance rate of mosaic embryos (Popovic et al., 2019). Taken together with our study result, it is suspected that the aneuploid cell decrease also occurred in their study and participated with the discordancy. These findings, both in humans and mice, suggest that aneuploid cell numbers are reduced during post-implantation embryonic development, a universal mammalian phenomenon.

It has been reported that embryos with low mosaicism (<40%) have high capability of continuing pregnancies (Munné et al., 2017). Previous report also showed difficulty to diagnose mosaic embryos by single embryo biopsy (Takahashi et al., 2021), and it is more difficult to evaluate details such as mosaicism level. Similarly in our present study, the results of karyotyping were different between multiple biopsy samples from the same embryo (shown in the Supplementary Table). However, we select mosaic embryos to be transferred or not by mosaicism level and the detail of chromosome with aneuploidy in clinical practice. Therefore, we considered the relationship between the level of mosaicism and embryo development, and tried to classify mosaic embryos by the maximum level of mosaicism of the embryo. We classified mosaic embryos with 20%–50% mosaicism as low-level mosaicism, and embryos with 50%–80% mosaicism as high-level mosaicism. Although there was no significant difference due to the small number of embryos, high-level mosaicism embryos obtained 8/12 (66.7%) of the embryos that changed mosaic to euploid during culture. As for culturability, 7/9 (77.8%) of the culturable embryos were high-level mosaicism. Furthermore, the number of mtDNA mutations tended to be lower in high-level mosaicism embryos than that in low-level mosaicism embryos, but the difference was also not significant (high-level vs. low-level; 1.00 ± 1.00 vs. 0.20 ± 0.33, *p* = 0.14). From those results, we could suggest high-level mosaicism embryos also have possibility to develop well after implantation, further studies are required to clarify this.

It should be noted that the mechanism underlying the decrease in aneuploid cell number remains elusive. Mitochondria possess critical functions in human embryonic development, such as ATP synthesis and OXPHOS. Disrupting OXPHOS and low ATP content can hinder oocyte maturation, along with chromosomal misalignment, fertilization, and implantation (Van Blerkom et al., 1995; Takeuchi et al., 2005). Based on these findings, we speculated that mitochondrial functions play an important role in post-implantation embryonic development and participate in aneuploid cell reduction.

mtDNA contains 13 protein-coding genes. These proteins are components of the mitochondrial OXPHOS enzyme complex (complexes I, III, IV, and V), which is involved in cellular ATP synthesis. *ND1-6* are critical for complex I, which is the starting point for OXPHOS. Protons are pumped into the mitochondria through complexes I, III (*CYTB*) and IV (*COI* and *COII*), and this electrochemical gradient is essential for the mitochondrial membrane potential and ATP synthesis in complex V (*ATPase6* and *ATPase8*). Based on these function of mtDNA, mitochondrial function is regulated by the amount of mtDNA, which controls the transcription of mitochondrial genes (Williams, 1986; Hock and Kralli, 2009). Therefore, a common method for evaluating mitochondrial function in a few cells is to determine the mtDNA-to-nuclear DNA ratio, called the mtDNA copy number assay (Malik and Czajka, 2013). Several previous reports have linked mtDNA copy number to embryo quality; however, the precise relationship remains unclear (Podolak et al., 2022b). Herein, although culturable embryos had lower mtDNA copy number, we found no significant difference. From our results, the mtDNA copy number did not seem to correlate with embryo culturability and reducing aneuploid cell. Our mtDNA copy number results showed large differences between samples derived from the same embryo. This may be related to the fact that most of the donated embryos were morphologically low grade. Furthermore, the reported non-symmetrical distribution of mitochondria among blastomeres (Podolak et al., 2022a) would also associate. This unequal division of mitochondria will be one of the disturbances of embryonic mtDNA copy number analysis and further innovation of the technologies to measure mtDNA copy number of embryos is required.

Owing to the generation of reactive oxygen species in mitochondria, lack of DNA protective histones, and limited mtDNA repair mechanisms, mtDNA has a higher number of mutations than nuclear DNA (Payne and Chinnery, 2015), and these mtDNA mutations can result in pathological dysfunctions (Schon et al., 2012). Although not all mtDNA mutations induce mitochondrial dysfunction, non-synonymous and highly heteroplasmic mtDNA mutations can impact mitochondrial function. Analysis of these mtDNA mutations can facilitate the prediction of mitochondrial function in limited cells. Therefore, we focused on mtDNA mutations to determine whether mitochondria are associated with human embryonic development.

In the present study, we demonstrate the number of non-synonymous and highly heteroplasmic mtDNA mutations in post-culture euploid embryos was significantly lower than that in post-culture aneuploid embryos. In oocyte, the mitochondrial membrane potential is made by protons via OXPHOS and was found to be associated with embryo aneuploidy and inhibition of apoptosis (Wilding et al., 2003; Takashima et al., 2021). A non-synonymous single-base mtDNA mutation in *COI* was reported to induce resistance to drug-induced apoptosis (Sun et al., 2015). mtDNA mutations in embryos may have same effect and influence the mitosis. These reports suggest that non-synonymous and highly heteroplasmic mtDNA mutations may influence unequal blastomere proliferation and the remaining of aneuploid cells.

In present study, the number of non-synonymous mtDNA mutations with ≥90% heteroplasmy was significantly lower in culturable embryos than that in non-culturable embryos. In previous report, pre-implantation development of human embryos is independent of the number of mtDNA mutations, and specific mtDNA mutations with low heteroplasmy does not affect implantation outcomes (Chatzovoulou et al., 2021). The number of non-synonymous and low heteroplasmic mtDNA mutations in our study also did not differ between the culturable and non-culturable embryos (data not shown). In addition, increased mtDNA mutations in mouse embryos did not impact blastocyst development, but did decline post-implantation development (Han et al., 2022). Taken together, these results suggest that non-synonymous and highly heteroplasmic mtDNA mutations at pre-implantation stage may relate with both of post-implantation karyotype and culturability. Furthermore, it was reported that even euploid embryos led to implantation failure in 30%, and clinical miscarriage in 10% (Forman et al., 2012). The mtDNA mutations may also relate with a part of these implantation failure and miscarriage which is independent with embryo karyotypes.

Our mtDNA evaluation of peri-implantation embryos with non-synonymous and highly heteroplasmic mtDNA mutations revealed that euploid and culturable embryos had fewer mtDNA mutations than aneuploid and non-culturable embryos. We concluded that the number of non-synonymous and highly heteroplasmic mtDNA mutations at pre-implantation stage would importantly influence post-implantation development by altering OXPHOS. Further investigation of this influence may enable us to predict post-implantation development.

Additionally, even in our small analysis of mtDNA mutation locations, karyotype and culturability differed significantly. Present result is supported by the findings of Ma et al., who reported that mtDNA mutations in OXPHOS protein-coding genes were related to failed embryonic development in mice (Ma et al., 2020). Furthermore, oocytes from older patients, often associated with failure of embryonic development, reportedly exhibit reduced *ND1* gene expression (Llonch et al., 2021). Another report on transcriptome differences according to the karyotype of embryos has highlighted the substantially upregulated expression levels of mitochondrial ATP synthase (Groff et al., 2019).

The number and location of mtDNA mutations, especially *ND1*, *COII*, *ATPase8*, *ND3*, and *CYTB*, may play a critical role in the mitochondrial function during post-implantation embryonic development. Although poorly understood, our results and those of previous studies suggest that specific mtDNA gene mutations may critically impact embryonic development and karyotype. Further human studies are required to clarify the relationship between mtDNA mutations and human embryonic development. The detailed establishment of this relationship could help to predict post-implantation embryonic development in more detail using the number and location of non-synonymous and highly heteroplasmic mtDNA mutations.

Nevertheless, the present study, which employed human embryos, had unsolvable limitations. Owing to ethical restrictions, most of evaluated embryos in this study were discarded and morphologically low-grade embryos. Although this restriction may influence our result, better quality embryos needed to be used for fertility treatment and our result may lead to the methods to use morphologically low-grade embryos. We also could not secure a large number of embryos and needed to evaluate post-implantation development using an *in vitro* culturing system without embryo transfer into the uterus. Pre-culture samples were biopsied from the TE, and post-culture samples were collected from the outgrowth of the *in vitro* cultured embryo including cells derived from ICM and TE. If we had biopsied the ICM from morphologically low-grade pre-cultured embryos, establishing subsequent culture might have been impossible. This unsolvable mismatch always occurs with the current PGT. In this study, we analyzed mutations of mtDNA. However, our primer set for selective amplification of mtDNA cannot amplify mtDNA with 4977bp deletion which is common and can be an aging marker of oocytes (Arnheim and Cortopassi, 1992). Therefore, we need to analyze the embryos with this deletion in next step. The relationship between mtDNA mutations and mitochondrial function has not been directly assessed, given that only a few cells can be obtained via TE biopsy. Accordingly, further innovations and advancements in this field are needed to address these limitations.

In conclusion, our findings revealed that some human mosaic embryos can develop into euploids, and suggested the number of aneuploid cells decreases in human mosaic embryos during post-implantation development. Furthermore, post-culture euploid and culturable embryos had fewer non-synonymous and highly heteroplasmic mtDNA mutations than aneuploid embryos and non-culturable embryos. Taken together, mitochondrial function may be one of important factor in reducing aneuploid cells and in the development of post-implantation human embryos. Further studies of this area may enable us to select better mosaic embryos for transfer, and may also enable us to transfer better embryos among euploid embryos, which could increase clinical pregnancy rates.

## Data Availability

The data presented in the study are deposited in the DDBJ repository, accession number “DRR491368-DRR491450” further inquiries can be directed to the corresponding author.
