# Supplementary material for: Mitochondrial DNA mutations can influence the post-implantation development of human mosaic embryos
Source: Front Cell Dev Biol. 2023 Aug 11;11:1215626. doi: 10.3389/fcell.2023.1215626 (PMC10451077; doi:10.3389/fcell.2023.1215626)
Supplement: Supplementary file 1 [file Table5.DOCX]

Supplementary Material

Mitochondrial DNA mutations can influence the post-implantation development of human mosaic embryos.

Akifumi Ijuin ^(1,2)^†, Hiroe Ueno ^(1)^†, Tomonari Hayama ^(1,3)^†*, Shunsuke Miyai ^(4)^, Ai Miyakoshi ^(1)^, Haru Hamada ^(1)^, Sumiko Sueyoshi ^(1,2)^, Shiori Tochihara ^(1)^, Marina Saito ^(1)^, Haruka Hamanoue ^(5)^, Teppei Takeshima^(1)^, Yasushi Yumura ^(1)^, Etsuko Miyagi ^(2)^, Hiroki Kurahashi ^(4)^, Hideya Sakakibara ^(3)^, Mariko Murase ^(1)^

†: These authors contributed equally to this work and share first authorship.

1. Reproduction Center, Yokohama City University Medical Center, Kanagawa, Japan

2. Dept. of OB & GYN, Yokohama City University School of Medicine Graduate School of Medicine, Kanagawa, Japan

3. Dept. of GYN, Yokohama City University Medical Center, Kanagawa, Japan

4. Division of Molecular Genetics, Institute for Comprehensive Medical Science, Fujita Health University, Aichi, Japan.

5. Dept. of Clinical Genetics, Faculty of Medicine, Yokohama City University, Kanagawa, Japan

# *Corresponding author:

Author name: Tomonari Hayama

E-mail address: [tommy_h@yokohama-cu.ac.jp](mailto:tommy_h@yokohama-cu.ac.jp)

# Supplementary Figures and Tables

## Supplementary Tables

Supplementary table 1. All data list
